# Recent approaches of antibody therapeutics in androgenetic alopecia

**DOI:** 10.3389/fphar.2024.1434961

**Published:** 2024-08-16

**Authors:** Su-Eon Jin, Jino Kim, Jong-Hyuk Sung

**Affiliations:** ^1^ Epi Biotech Co., Ltd., Incheon, Republic of Korea; ^2^ New Hair Plastic Surgery Clinic, Seoul, Republic of Korea

**Keywords:** androgenetic alopecia, therapeutic antibody, prolactin receptor, interleukin-6 receptor, C-X-C motif chemokine ligand 12, dickkopf 1

## Abstract

Therapeutic antibodies (Abs) have been anticipated as promising alternatives to conventional treatments such as topical minoxidil and oral finasteride for androgenetic alopecia (AGA). Due to the high molecular weight of typical Abs, the half-life of subcutaneous Abs exceeds 2 weeks, allowing an administration intervals of once a month or longer. Direct injection into the areas of hair loss is also feasible, potentially enhancing treatment efficacy while minimizing systemic side effects. However, therapeutic Abs are rarely developed for AGA therapy due to the requirement to be responsiveness to androgens and to exist in the extracellular fluid or cell surface surrounding the hair follicle. In this review, we introduce recent progress of antibody therapeutics in AGA targeting the prolactin receptor, Interleukin-6 receptor, C-X-C motif chemokine ligand 12, and dickkopf 1. As therapeutic Abs for AGA are still in the early stages, targets need further validation and optimization for clinical application.

## 1 Introduction

Androgenetic alopecia (AGA), also referred to as male pattern hair loss (MPHL), is the most common phenotype in hair loss caused by excessive androgen responsiveness mainly based on hormone imbalance, genetic familial history, and unknown etiology (Price, 1999; Phillips et al., 2017; Miguel Dominguez-Santas et al., 2022). In MPHL, hair miniaturization and shedding occur progressively in the frontal and vertex scalp regions by androgens such as dihydrotestosterone (DHT), leading to bitemporal hair thinning. Compared with MPHL, female pattern hair loss (FPHL) is more diffused in the vertex scalp region, irrespective of frontal hair line (Supsrisunjai and Thuangtong, 2016; Nicholas Sadgrove et al., 2023).

Therapeutic Abs have been developed over 150 years, and was awarded the first Nobel prize in 1901 for serum therapy of diphtheria (Ebrahimi and Samanta, 2023). Muromonab-CD3 (Orthoclone, OKT3, for acute transplant rejection) is the first monoclonal Ab (mAb) approved by the Food and Drug Administration (Todd and Brogden, 1989). Other therapeutic Abs have been also approved for severe human disorders including infection, autoimmunity, and cancer, as first-line mono therapeutics or combinations (Sharma et al., 2023). These therapeutic Abs are beneficial for their high specificity, low inherent variability, high lot-to-lot consistency, and predictable duration of action, though they have disadvantages including high complexity for Ab creation, and complicated technology integration (Chames et al., 2009; Nagarajan et al., 2014).

Diverse strategies for therapeutic Abs have been developed for alopecia areata (AA) therapy (Renert-Yuval and Guttman-Yassky, 2017). For example, dupilumab which was first developed for skin allergy improved type 2 T-cell immune response in AA (Guttman-Yassky et al., 2022). Secukinumab also showed efficacy and safety in patients with extensive AA (Guttman-Yassky et al., 2018). In general, Abs targeting cytokines resulted in promising treatment options for AA (Maciej Stępień and Anczyk, 2023). However, there are only few therapeutic Abs for AGA (Table 1). For example, prolactin receptor (PRLR) is on-going in phase 2, a proof-of-concept study (NCT06118866) (Heilmann-Heimbach et al., 2020) and interleukin-6 receptor (IL-6R) (Vidon et al., 2014) has been clinically reported with two patients. C-X-C motif chemokine ligand 12 (CXCL12) (Zheng et al., 2024) and dickkopf-1 (DKK1) (Fawzi et al., 2016) are also approached as targets for AGA in nonclinical trials. Target molecules for AGA should be validated to ensure the therapeutic efficacy from an integrated perspective. In this review, we introduce therapeutic Abs for AGA therapy in clinical trial and non-clinical research stages as mentioned above, together with the underlying molecular mechanism. Although Ab development still remains challengeable in the early process, therapeutic Abs can be promising as a safe and efficient medications for AGA.

**TABLE 1:** Antibody therapy for AGA.

Target gene	Ligand or receptor	Localization in hair/skin	Current development status	Outcome/mechanism	Reference
PRLR	PRL	DP, ORS	Phase II	PRLR Abs showed increased hair density to 14 hairs/cm2 in 12 male patients Hair cycle regulation	(Langan et al., 2010), (Chime Biologics, 2023)
IL-6R	IL-6	DP, ORS	Case report	Tocilizumab resulted in hair regrowth in the male patients suffering from AGA AR in DPCs increased IL6 secretion to inhibit ORS cells	(Vidon et al., 2014), (Kwack et al., 2012)
CXCL12	CXCR4	DF	Nonclinical	CXCL12 Ab increased hair growth in testosterone-induced AGA mice Inhibited AR activation in DP and reduced inflammation surrounding hair follicle	(Zheng et al., 2024), (Zheng et al., 2022)
Dkk1	LRP5, LRP6	DF, DP, ASC	Nonclinical	DKK1 Ab protected the survival of ORS cells inhibited by androgens	(Choi et al., 2024), (Kwack et al., 2008)

## 2 Advantage of Ab therapy in AGA

### 2.1 Current treatment options

Although topical minoxidil and oral finasteride have been approved as medications for AGA therapy, the inconsistent efficacy and adverse effects are still an issue among patients (Nestor et al., 2021; Kaiser et al., 2023). Consequently, topical application of finasteride had been approved and marked for AGA patients, followed by dutasteride. Finjuve Spray is considered a safer and more convenient way to get the same benefits minimizing these issues. In clinical trials, the Finjuve-group showed blood concentration levels of only one-hundredth of the oral form after 24 weeks of treatment (Caserini et al., 2014; Todeschini et al., 2022). Furthermore, developing a long-lasting medicine is crucial to improve the effectiveness and convenience of hair loss treatments. For instance, a long-acting injectable formulation of finasteride, using lipid nanoparticle, showed pharmacological effects and achievability with monthly administration (Wook Kang et al., 2023). However, long-acting formulation still exhibited systemic side effects and is currently in the early stage, phase 1 or two clinical trial (Ahmed et al., 2023).

### 2.2 Therapeutic advantages of Abs in AGA treatment

mAb therapy was first administrated *via* intravenous (i.v.) route, has shifted towards to subcutaneous (s.c.) injections in order to be a more patient-friendly approach (Jiskoot et al., 2022). Therefore, pharmacokinetic and distribution of Ab drugs after s.c. injection has been reported, which showed delayed tmax and longer half-life compared with i.v. (Sanchez-Felix et al., 2020). Moreover, s.c. delivery is safe, effective, and valued by patients.

**FIGURE 1:**
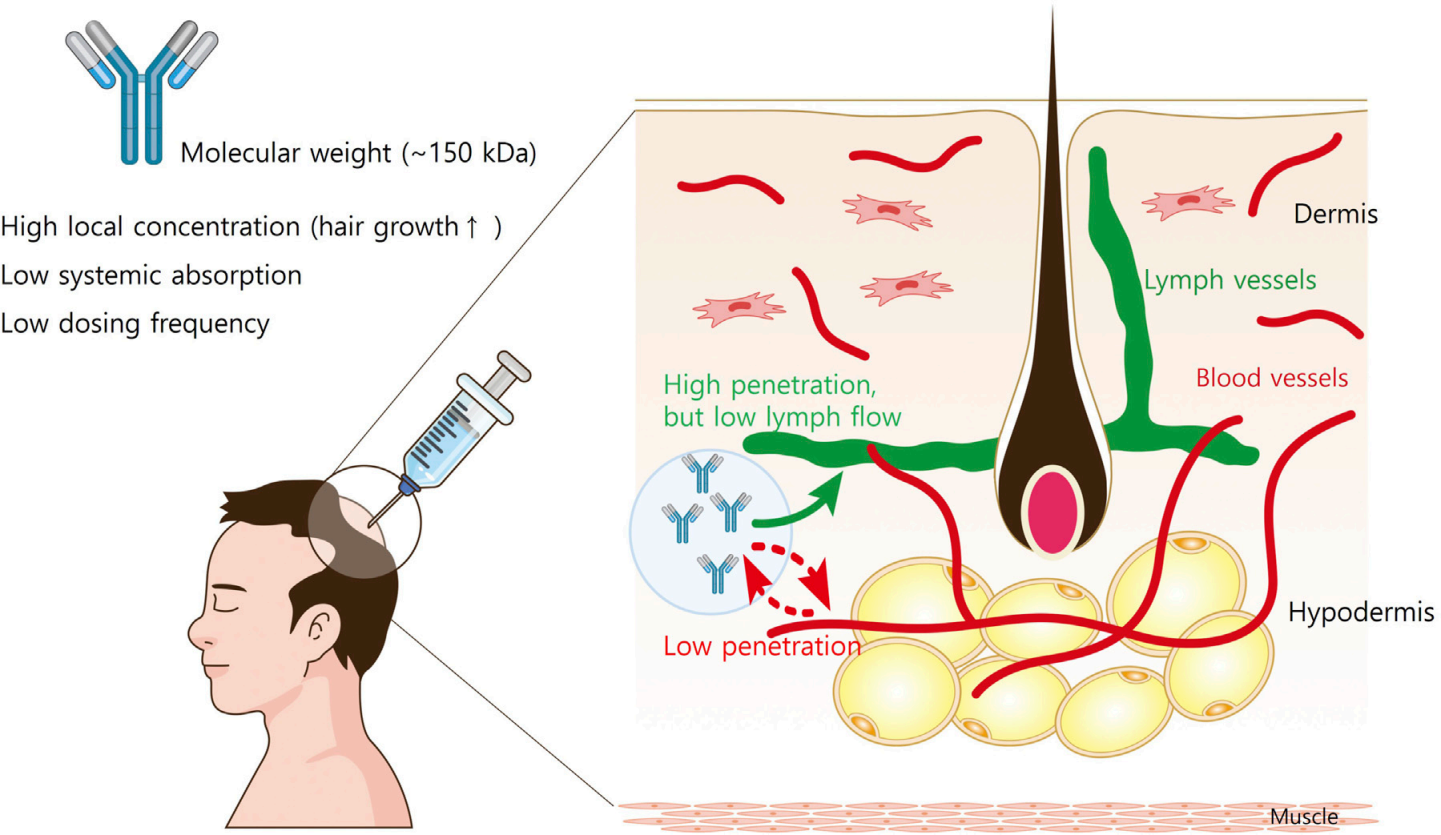
The structure of the skin and the movement of subcutaneously administered mAbs. The hypodermis consists of adipose and connective tissues interspersed with blood and lymphatic vessels. The connective tissues of the hypodermis are made up of highly polymerized macromolecular networks, and the vascular capillaries in the hypodermis are impermeable to large molecules (>50 kDa). Therefore, it is generally assumed that reaching from the hypodermis to systemic circulation results from lymphatic drainage for most mAbs. However, lymph flow is slower than 0.2% of blood flow, the systemic absorption of mAbs from injection site is limited.

The structure of the skin affects the movement of s.c.-administered mAbs. The hypodermis consists of adipose and connective tissues interspersed with blood and lymphatic vessels (Figure 1). The connective tissues of the hypodermis are made up of highly polymerized macromolecular networks, and move through the hypodermis *via* fluid flow-driven convection or non-convective diffusion (Davis et al., 2024). Given that the vascular capillaries in the hypodermis are impermeable to large molecules (>50 kDa), it is generally assumed that reaching from the hypodermis to systemic circulation results from lymphatic drainage for most mAbs (Porter and Charman, 2000). While mAb transcytosis through the blood capillary endothelial cell into circulation is possible, drainage *via* lymphatic capillaries represents the primary mechanism for mAb transport out of the interstitial space at the injection site (Zhou and Theil, 2015). However, lymph flow is significantly slower than blood flow (0.2% of blood flow), which contributes to the prolonged peak time (T_max_ = 2–14 days) observed in mAb absorption following s.c. administration (Charman and Stella, 1992).

Since the molecular weight of a typical Ab is very high (∼150 kD), many s.c. Ab medications are administered at regular intervals, often monthly to bimonthly (Ovacik and Lin, 2018). In addition, direct injection of Ab medications into the hair loss areas leads to superior treatment efficacy while minimizing the systemic side effects (Liu, 2018). Therefore, therapeutic Abs administered *via* s.c. injection are promising for AGA therapy.

## 3 Current therapeutic Abs for AGA therapy

Therapeutic Abs are rarely developed for AGA therapy due to the requirement to be responsiveness to androgens and to primarily exist in the extracellular fluid or cell surface surrounding the hair follicle. However, therapeutic Abs targeting the PRLR (Foitzik et al., 2006; May et al., 2019), IL-6R (Sheppard et al., 2017; Walker et al., 2021), CXCL12 (Zheng et al., 2022; Zheng et al., 2024), and DKK1 (Fawzi et al., 2016; Choi et al., 2024) have been reported to improve AGA both in clinical and non-clinical settings.

### 3.1 PRLR antibody

PRL has been considered as a hormonal hair growth regulator, also functioning in angiogenesis, adipogenesis, and immune response beyond lactation (Grymowicz et al., 2020; Heilmann-Heimbach et al., 2020). It is a central neurohormone in hypothalamic-pituitary axis, released from the pituitary gland stimulated by prolactin releasing hormone. The pilosebaceous unit, another epidermal derivative, has emerged as a prominent, PRLR expressing, nonclassical PRL target organ. PRL mediates psoriasis, AGA, and stress-related dermatoses in the skin.

Human scalp hair follicles expressed PRL and PRLR, which served as an autocrine and/or paracrine mediators of apoptosis-driven hair follicle regression. PRL and PRLR were upregulated during the hair regression period, and high-dose PRL (400 ng/mL) resulted in a significant inhibition of hair shaft elongation and premature catagen development, along with reduced proliferation and increased apoptosis of hair bulb keratinocytes (Foitzik et al., 2006). However, there is a controversial report suggesting that moderately elevated PRL levels may not significantly contribute to diffuse hair loss (Lutz, 2012). Additionally, PRL has been shown to exert gender- and/or site-specific effects on the human hair follicle (Langan et al., 2010). Of note, HMI-115 is an mAb targeting PRLR (May et al., 2019; Castro et al., 2023) currently in an on-going clinical trial at phase 2 for AGA (NCT06118866) (Barrie, 2024). At phase 1b, HMI-115 showed increased hair density to 14 hairs/cm^2^ of non-vellus hair count in 12 male patients (Chime Biologics, 2023).

PRL and its receptor are also expressed in the murine hair follicle epithelium, showing hair cycle-dependent expression (Foitzik et al., 2003). PRL has shown to delay hair regrowth in mice and is involved in hair cycle regulation, rather than other hormonal factor regulation (Craven et al., 2006). A neutralizing PRLR Ab treatment *in vivo* stimulated hair regrowth in female mice (Otto et al., 2015). Compared with peptide-derived PRLR antagonists, the PRLR Ab exhibits several advantages such as higher potency, noncompetitive inhibition of PRLR signaling, and a longer half-life, which enhances its suitability in long-term animal studies.

### 3.2 IL-6R antibody

IL-6 is a key cytokine of immune modulation, affecting autoimmune diseases, chronic inflammatory bowel disease, vasculitis, and cancer (Calabrese and Rose-John, 2014). Of interest, IL-6 is involved in androgen-driven alteration to the autocrine and paracrine factors, and is induced by DHT in dermal papilla (DP) of balding scalps (Kwack et al., 2012). IL-6R and glycoprotein 130 were also expressed in follicular keratinocytes, and IL-6 has shown to reduce the hair shaft elongation and the matrix cell proliferation in cultured human hair follicles. Moreover, IL-6 injection into the hypodermis of mice resulted in premature onset of catagen, ultimately inhibiting the hair growth as a paracrine factor from the DP.

Tocilizumab is an anti-human IL-6R mAb to treat rheumatoid arthritis and cytokine release syndrome (Sheppard et al., 2017). It inhibits the binding of IL-6 to the receptor, reducing pro-inflammatory activity by competing with soluble and membrane-bound forms of IL-6R (Jones and Hunter, 2021; Rose-John et al., 2023). As IL-6 is involved in inflammation and immune stimulation, it was primarily investigated for AA patients by disrupting the IL-6 signaling, leading to hair regrowth (Walker et al., 2021). Interestingly, repeated treatment of tocilizumab resulted in hair regrowth after several months in the male patient suffering from AGA (Vidon et al., 2014).

### 3.3 CXCL12 antibody

CXCL12 and its receptor CXCR4, which are highly expressed in the skin, are associated with various cutaneous diseases. CXCL12 plays multifaceted roles in cellular migration, tissue homeostasis, and wound healing. We first investigated the involvement of CXCL12 in the hair cycle regulation, and found that CXCL12 is highly expressed in dermal fibroblasts (DFs) and its level was elevated throughout the catagen and telogen phases of the hair cycle (Zheng et al., 2022). Hair loss was induced by recombinant CXCL12 therapy in hair organ culture, which also delayed the telogen-to-anagen transition and decreased the hair length in the animal model. On the contrary, the suppression of CXCL12 using a neutralizing Ab triggered the telogen-to-anagen transition in the animal model. Similarly, pharmacological inhibition of CXCR4 increased hair growth, which indicates that CXCL12/CXCR4 pathway inhibitors are promising treatment options for promoting hair growth.

**FIGURE 2:**
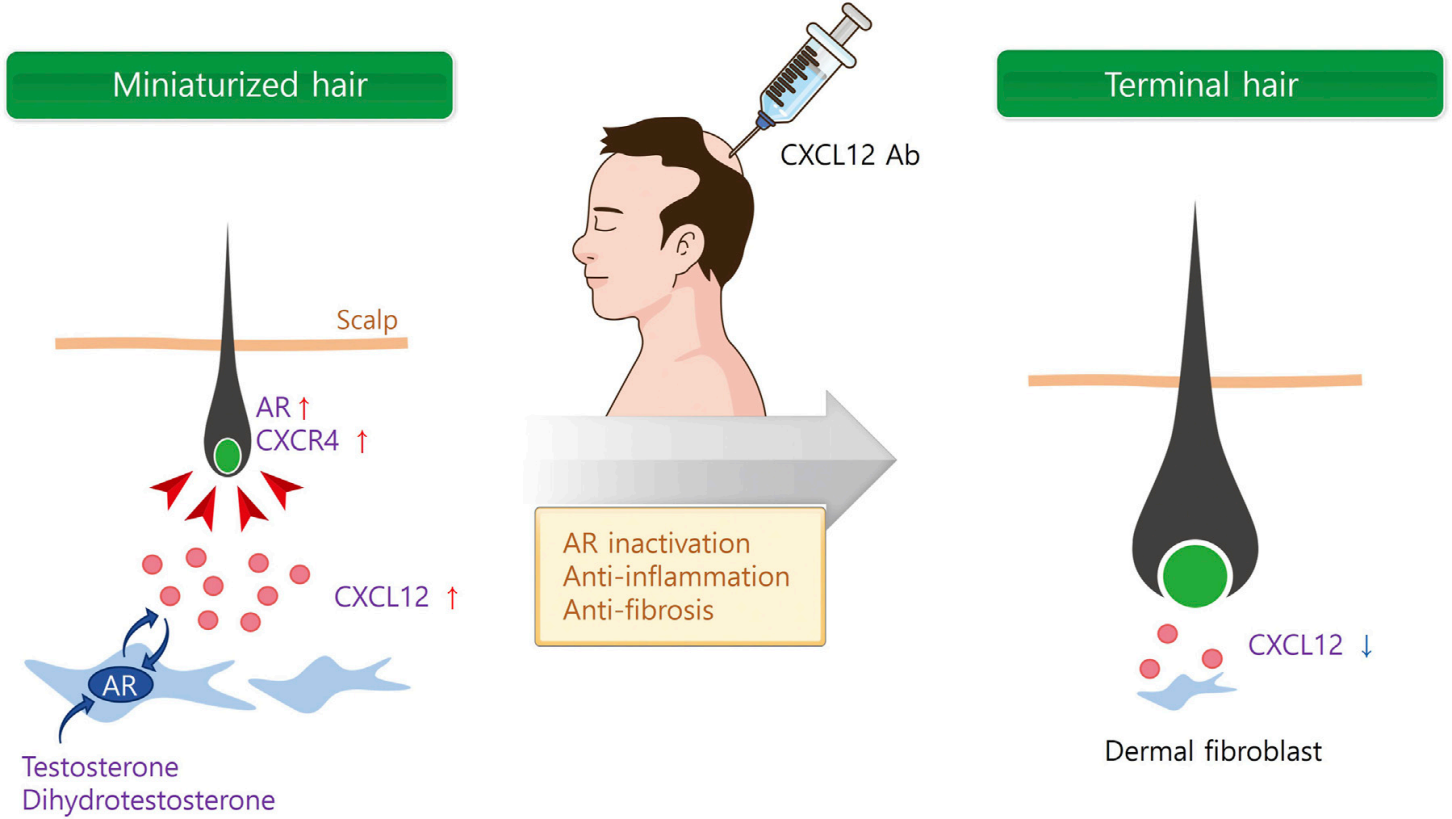
Multi-functional role of CXCL12 for AGA therapy. Androgen hormones secrete CXCL12 from dermal fibroblasts (DF) *via* androgen receptor (AR). Secreted CXCL12 activates AR in DF and dermal papilla cells (DPCs), and miniaturized hair follicle. On the contrary, CXCL12 Ab inactivates AR in DF and DPCs, and inhibits inflammation and fibrosis surrounding hair follicle.

In addition to androgen hormones, inflammatory mediators like CXCL12 were significantly increased in the scalps of AGA patients (Michel et al., 2017). It is of interest that CXCL12 expression appeared to interact with the androgen signaling pathway. For instance, testosterone and DHT upregulated CXCL12/CXCR4 expression through the androgen receptor (AR) (Chodari et al., 2016; Asbelaoui et al., 2024). Therefore, we further investigated whether a CXCL12 neutralizing Ab is effective for AGA treatment. The mRNA and protein expressions of CXCL12 were high in the DFs of mouse skin after testosterone and DHT treatment (Zheng et al., 2024). Testosterone and DHT significantly delayed the hair growth, whereas s.c. injection of CXCL12 Ab significantly increased hair growth in the AGA model (Figure 2).

We further examined the underlying molecular mechanism of CXCL12 in the process of improving AGA models. Of note, AR is primarily expressed in both DFs and DP in the skin, and co-localized with CXCL12 in DFs (Michel et al., 2017). Therefore, DFs were incubated with various concentrations of DHT or testosterone (1–100 nM), resulting in a significant increase in both CXCL12 mRNA and protein levels compared to control. Androgens translocated AR to the nucleus in DFs, while AR-KO significantly decreased the CXCL12 secretion after androgen treatment. These results indicate that androgens induce CXCL12 expression in DFs and AR is involved in the CXCL12 expression.

In addition to androgens, the activation of an inflammatory response towards the pilosebaceous unit may play a role in the development and progression of AGA, with perifollicular inflammation and fibrosis reported in histologic sections (Trüeb et al., 2010; Valdebran et al., 2020). Recent research has proved that the CXCL12/CXCR4 axis plays a critical role in the inflammatory pathway due to its chemotaxis to inflammatory cells (Lu et al., 2024). Blocking the chemotaxis of inflammatory cells by CXCL12 in the scalp may inhibit and alleviate the inflammatory response to cure AGA. Furthermore, the CXCL12/CXCR4 axis is also involved in fibrosis in various organs, and CXCL12/CXCR4 inhibition showed a promising approach in the fight against fibrosis (Wu et al., 2023). Therefore, it is reasonable to assume that CXCL12 Ab cures AGA through AR inactivation, anti-inflammation, and prevention of fibrosis (Figure 2).

### 3.4 DKK 1 antibody

DKK1 expression has been highly determined in AGA patients as well as AA patients (Fawzi et al., 2016; Choi et al., 2024). DKK1, primarily expressed in adipose-derived stem cells (ASCs), tends to decrease following adipogenic differentiation (Christodoulides et al., 2006). We first examined the involvement of DKK1 in AA, and found that DKK1 is secreted from the ASCs of AA patients. Also, discovered that neutralizing Abs for DKK1 is effective in AA treatment. Specifically, subcutaneous injection of DKK1 Abs reduced the area of hair loss in AA, and CD8^+^ cells in the skin (Choi et al., 2024).

Circulating androgens enter the follicle, and bind to the AR within the DP cells to activate or repress target genes. Kwack et al., screened DHT-inducible genes in balding DP cells by cDNA microarray and found that DKK1 is one of the most upregulated genes (Kwack et al., 2008). They also showed that neutralizing antibody against DKK1 was protective in the survival of ORS cells inhibited by androgens. The protective effect of anti-DKK1 antibody on DHT-induced cell death was examined in hair organ culture.

DKK1 is an endogenous pathogenic inhibitor of Wnt/β-catenin signaling in AGA (Premanand and Reena Rajkumari, 2018), which induced the anagen-to-catagen transition in the hair growth cycle (Mahmoud et al., 2019). Elevated androgen and AR in AGA also modulated Wnt/β-catenin signaling pathway in the DPCs of the balding scalp (Premanand and Reena Rajkumari, 2018). DKK1 signaling was mediated by the interaction of low-density lipoprotein receptor-related protein 6, and activated proapoptotic protein Bax to induce apoptosis in the ORS cells (Kwack et al., 2008). For this reason, DKK1 neutralizing Ab can be developed for AGA treatment.

## 4 Further consideration for therapeutic Ab development

The advantages of Abs and four therapeutic targets for in AGA have been described. However, developing a novel Ab therapy for AGA faces several challenges, including cell penetration, immunogenicity, and formulation.

### 4.1 Penetration into the cell and intracellular AR localization

Large number of diseases involve cytosolic targets, and designing Abs able to efficiently reach intracellular compartments would expand the antibody-tractable conditions. However, Abs are primarily effective as a therapeutic modality for interfering with targets in the extracellular space or at the cell membrane. Unlike small-molecule drugs that are capable of inhibiting some intracellular targets, Abs have high molecular weight and can target the extracellular molecules and have low volumes of distribution in humans (Salgado and Cao, 2020). It is therefore highly desirable to efficiently deliver antibodies intracellularly. Several studies, primarily in cultured cells, have shown the feasibility of facilitating antibodies’ cellular internalization (Herrmann et al., 2019).

As AR is primarily involved in AGA progression, there are many evidence supporting that the inhibition of AR activation cures AGA using siRNA, miRNA, and small molecules (Bielska et al., 2022; Moon et al., 2023). However, AR is an intracellular nuclear receptor, there is no Abs to target AR in AGA therapy to date. Of interest, bispecific Abs targeting the N-terminal domain of AR have been developed for prostate cancer (Goicochea et al., 2017). Bispecific Abs entered human LNCaP prostate cells, accumulated in the nucleus, and inhibited the growth of prostate cancer cells under androgen-stimulated conditions. Therefore, these bispecific Abs can be used for AGA treatment.

### 4.2 Functional role of AR in DPCs, hair follicle stem cells, and DFs

Hair follicle consists of diverse type of cells such as DPCs, ORS cells, and hair follicle stem cells (HFSCs). Among these, DPCs and ORS cells interact with each other to generate new hair follicles and to influence hair type. AR activation in DPCs regulates ORS cells by modifying the paracrine factors produced by DPCs (Hamada and Randall, 2006). Reduction in both DP volume and cell number in AGA suggest that AR expression within the DP plays a key role in altering the hair size in response to androgens (Elliott et al., 1999). Therefore, targeting interaction between DPC and ORS cells is important for AGA therapy, and Abs for PRLR and IL-6R primarily mediate hair growth by directly targeting DPCs and ORS cells.

Recently, the functional role of AR in HFSCs has been reported. For instance, androgens deregulate DPC-secreted factors through AR, which inhibits normal HFSC differentiation by suppressing the canonical Wnt signaling pathway in AGA (Leiros et al., 2012). Additionally, it has been reported that AR directly affects HFSCs by antagonizing the Wnt/β-catenin signaling pathway. Inhibition of AR enhances the effects of β-catenin activation, thereby promoting HF proliferation and differentiation (Kretzschmar et al., 2015).

Interestingly, AR is highly expressed in DFs in addition to DPCs in human (Jacobson et al., 1995; Clocchiatti et al., 2018). However, functional role of AR in DFs has not been fully reported in the AGA progression. We first investigated the functional expression of AR and CXCL12 in DFs, and found that they form positive feed-back loop to regulate hair cycle. Androgens increased CXCL12 expression and CXCL12 induced AR to inhibit hair growth in AGA (Asbelaoui et al., 2024; Zheng et al., 2024). Therefore, inhibition of AR in DFs through Ab therapy is a promising novel therapeutic target for AGA.

### 4.3 Immunogenicity

Early studies on protein vaccine development provided evidence that the s.c. route of administration typically elicits a more robust immunogenic response compared with the i.v. route (De Groot and Moise, 2007; Pollard and Bijker, 2021). It is hypothesized that, mAb is trafficked first through local lymphatic capillary beds and then into larger lymphatic vessels, which may potentially result in increased antigen presentation by dendritic cells. However, to our knowledge, publicly available clinical datasets where immunogenicity is reported for both i.v. and s.c. routes of administration are generally not supportive of this hypothesis. Two review articles have summarized immunogenicity for 11 therapeutic proteins (Hamuro et al., 2017; Xu et al., 2023). Of these, the incidence of anti-drug antibodies was comparable between the s.c. and i.v. products for 10 of the 11 therapeutic proteins.

## 5 Discussion

The efficacy and pharmacokinetic profiles of conventional approved AGA therapy such as the topical minoxidil and oral finasteride remain limited, emphasizing the need of alternative therapeutic medications. Therapeutic Abs can be promising candidates in company with a long-lasting characteristic and enhanced safety *via* s.c. administration route. Inhibiting overexpression of AR and 5α-reductase by Abs can be a primary strategic option to manage AGA reducing off-target effects, however, they are intracellular target which poses difficulty in neutralization. Therefore, cell surface and secreted proteins surrounding hair follicle are the targets for AGA, and Abs for PRLR, IL-6R, CXCL12, and DKK1 have been developed and characterized. Strategic development of therapeutic Abs is necessary and, new targets should be validated and optimized for AGA treatment.
